# Development of microscopic polyangiitis-related pulmonary fibrosis in a patient with autoimmune pulmonary alveolar proteinosis

**DOI:** 10.1186/1471-2466-14-172

**Published:** 2014-11-04

**Authors:** Yuhei Kinehara, Hiroshi Kida, Yoshikazu Inoue, Masaki Hirose, Akihiko Nakabayashi, Yoshiko Takeuchi, Yoshitomo Hayama, Kiyoharu Fukushima, Haruhiko Hirata, Koji Inoue, Toshiyuki Minami, Izumi Nagatomo, Yoshito Takeda, Toshiki Funakoshi, Takashi Kijima, Atsushi Kumanogoh

**Affiliations:** Department of Respiratory Medicine, Allergy and Rheumatic Diseases, Osaka University Graduate School of Medicine, 2-2 Yamada-oka, Suita, Osaka, 565-0871 Japan; National Hospital Organization Kinki-Chuo Chest Medical Center, 1180 Nagasone-Cho, Kita-Ku, Sakai, Osaka, 591-8555 Japan; SenriSaiseikai Hospital, 1-1-6 Tsukumodai, Suita, Osaka, 565-0862 Japan

**Keywords:** Pulmonary alveolar proteinosis, Pulmonary fibrosis, Myeloperoxidase antineutrophil cytoplasmic antibody

## Abstract

**Background:**

Autoimmune pulmonary alveolar proteinosis (aPAP) is a rare lung disease caused by the autoantibody against granulocyte-macrophage colony stimulating factor (GM-CSF). The clinical course of aPAP is variable; in severe cases, patients develop lethal respiratory failure due to pulmonary fibrosis. However, the pathogenesis of pulmonary fibrosis in aPAP has never been delineated.

**Case presentation:**

Here, we describe a rare case of aPAP that was subsequently complicated by microscopic polyangiitis-related pulmonary fibrosis. The patient was a 75-year-old Japanese man diagnosed with aPAP based on the crazy-paving appearance on high-resolution computed tomography (HRCT), “milky” appearance of broncho-alveolar lavage fluid (BALF), and elevated serum levels of the anti-GM-CSF antibody. The patient was followed-up without aPAP-specific treatment for 3 years. During this period, both hematuria and proteinuria appeared; in addition, serum myeloperoxidase (MPO)-anti-neutrophil cytoplasmic antibody (ANCA) turned positive and increased markedly. The second BAL performed one year after the diagnosis, showed that the “milky” appearance had resolved. The HRCT showed that fibrotic changes had developed and that the crazy-paving appearance had disappeared. These data suggest an association between pulmonary fibrosis that developed during the natural course of aPAP and ANCA-related systemic vasculitis.

**Conclusion:**

This is the first case report that suggests the existence of a pathogenetic relationship between ANCA-associated systemic vasculitis and aPAP-related pulmonary fibrosis. The link between ANCA-associated systemic vasculitis and aPAP-related pulmonary fibrosis requires further investigation.

## Background

Autoimmune pulmonary alveolar proteinosis (aPAP), which causes 90% of all PAP cases, is an autoimmune disease caused by the presence of an autoantibody against granulocyte-macrophage colony stimulating factor (GM-CSF) [1]. The suppression of GM-CSF signaling by anti-GM-CSF autoantibody disrupts the surfactant catabolism, resulting in the accumulation of surfactant lipids and proteins in pulmonary alveolar macrophages and alveoli. The clinical course of aPAP is variable. In the contemporaneous cohort of 223 Japanese aPAP patients, 3 patients (1.4%) were complicated by severe respiratory failure due to pulmonary fibrosis [2]. And so far, there have been, at least, 5 case reports, which highly suggested the pathogenetic relationship between aPAP and pulmonary fibrosis [3–7].

The pathogenesis of pulmonary fibrosis in aPAP is unknown. It has been hypothesized that the retention of lipoproteinaceous material in the alveoli, silica exposure, and/or superimposed pulmonary infections induces damage to cells lining the alveoli and causes pulmonary fibrosis in aPAP patients [5]. In rats, the overexpression of GM-CSF in the lung by adenovirus-vector leads to pulmonary fibrosis, suggesting an inconclusive relationship between GM-CSF therapy and pulmonary fibrosis in patients with aPAP [8].

Anti-neutrophil cytoplasmic antibody (ANCA) is a sensitive and specific marker for ANCA-associated systemic vasculitis, as observed in granulomatosis with polyangiitis (GPA), microscopic polyangiitis (MPA), “idiopathic” necrotizing crescentic glomerulonephritis and allergic granulomatous angiitis [9]. In vitro and in vivo studies provide compelling evidence that ANCA play a critical role in the pathogenesis of ANCA-associated systemic vasculitis [10]. Pulmonary fibrosis due to alveolar capillaritis is a common complication in ANCA-associated systemic vasculitis. However, ANCA-related pulmonary fibrosis has never been reported in association with aPAP.

## Case presentation

**Figure 1:**
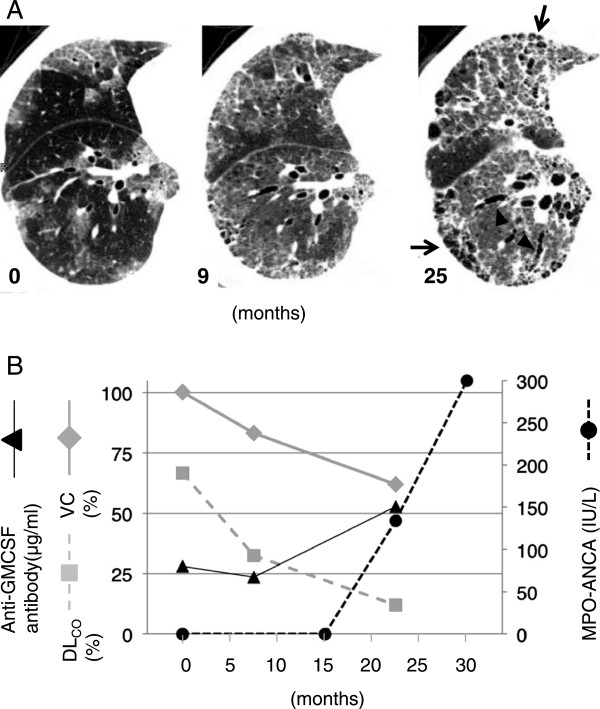
**During the clinical course of aPAP in this patient, HRCT images showed increased evidence of pulmonary fibrosis: reticular opacity (arrows) and traction bronchiectasis (arrowheads). (A)** Serum MPO-ANCA testing became positive and then increased significantly (circle), while the results of pulmonary function tests, including vital capacity (VC, diamond) and CO diffusing capacity (DLCO, square), deteriorated. Serum anti-GM-CSF antibody concentrations kept high and increased **(B)**.

A 75-year-old Japanese man, who self-identified as a chronic smoker (30 pack-years), was referred to our hospital because of a cough, which had been continued for 2 months, and an abnormal chest X-ray. He had no significant past medical history. He was an owner of a liquor shop and had no prior exposure to harmful dust. The physical examination revealed fine crackles bilaterally over the lower lung. The laboratory findings showed elevated levels of lactate dehydrogenase (376 U/L; normal range, 120-242), Krebs von den Lungen-6 (17330 U/mL; normal range, 0-500), surfactant protein-D (293.0 ng/mL; normal range, 0–110), carcinoembryonic antigen (12 ng/mL; normal range, 0–5.0), and CYFRA21.1 (cytokeratin-19 fragments) (26.5 ng/mL; normal range, 0–20.0) in the serum. Serum antinuclear antibody titer was 1:40 (normal range, <1:40), and myeloperoxidase (MPO)-ANCA testing was negative. The chest X-ray showed diffuse bilateral ground-glass opacities, and high-resolution computed tomography (HRCT) showed a crazy-paving appearance (Figure 1A). Bronchoscopy was performed; the broncho-alveolar lavage fluid (BALF) from the left lingura (B^5^) showed a typical “milky” appearance. The total cell count in BALF was 320/μL. Differential cell count revealed 50% alveolar macrophages, 36% lymphocytes, 12% neutrophils, 2% eosinophils. Pathological examination of a transbronchial lung biopsy sample revealed periodic-acid-Schiff stain-positive material along the alveolar wall. After the serum anti-GM-CSF autoantibody concentration (28 μg/mL; normal range, <0.5) was confirmed and the comorbidity of hematological diseases, such as chronic myelocytic leukemia, myelodysplastic syndrome, or monoclonal gammopathy of undetermined significance, were excluded, the patient was diagnosed with aPAP.

Because the patient refused any specific treatment for aPAP, including whole-lung lavage and GM-CSF inhalation therapy, he was followed in the outpatient department. Nine months later, his cough had worsened and he began to experience dyspnea upon exertion, which was classified using the modified Medical Research Council Dyspnea Scale (mMRC) as grade 2. The chest HRCT obtained at this visit showed that the crazy-paving pattern had been replaced by fibrotic changes, i.e., subpleural honeycombing and traction bronchiectasis (Figure 1A). In contrast to the results obtained at the time of diagnosis, the BALF was transparent, without a “milky” appearance. Serum anti-GM-CSF autoantibody concentration was 23.5 μg/mL. Surprisingly, serum MPO-ANCA testing was positive (134 IU/L) and subsequently continued to rise (Figure 1B). In accordance with the fibrotic changes seen on HRCT images, the patient’s respiratory failure had worsened since diagnosis (Figure 1B). The patient also showed signs of hematuria and proteinuria. Although neither kidney nor lung biopsies were performed because of the patient’s general condition, the findings presented above met the most recent criteria for MPA [11]. Because the patient refused steroid and immunosuppressant treatment, he has been followed-up for MPA without any specific therapy.

Many studies have shown that ANCA production is key to the pathogenesis of MPA. ANCA is known to activate neutrophils and allow their accumulation at the endothelial portion of a vasculitic lesion [12]. ANCA is also an excellent diagnostic marker of MPA with specificity of 96.3-99.1% [13]. Based on these findings, the European League against Rheumatism and the American College of Rheumatology established new criteria that emphasized serum ANCA in the diagnosis of MPA [14]. A new criterion of MPA, proposed by Watts, lays more emphasis on the role of ANCA in MPA diagnosis, wherein histopathological signs of vasculitis are not essential for the diagnosis [11]. Our case met these criteria due to the patient’s high level of MPO-ANCA and the associated urinary findings.

There are several reports of pulmonary fibrosis that developed in patients with aPAP. In these cases, as in ours, the features of PAP disappeared as pulmonary fibrosis progressed [5, 7]. As Luisetti et al. mentioned, it is not possible for us to exclude that some subjects diagnosed with diffuse fibrotic lung disease actually represented the end-stage evolution of a previous pulmonary alveolar proteinosis process [7]. If a patient’s BALF or HRCT does not show a typical PAP appearance at the time of admission, serum GM-CSF autoantibody is not usually measured. Thus, the link between PAP and pulmonary fibrosis must be explored. In this report, we suggest the role of ANCA-associated systemic vasculitis in the pathogenesis of aPAP-related pulmonary fibrosis. We sought to verify the existence of ANCA-associated systemic vasculitis in patients with aPAP-related pulmonary fibrosis, because in these cases, steroids or immunosuppressants (including rituximab) that are not usually used for the treatment of aPAP may be effective for the treatment of pulmonary fibrosis [15]. There is one reported case of secondary PAP with high levels of MPO-ANCA [16]. Further research will be necessary to elucidate the link between aPAP and ANCA-associated systemic vasculitis. Serum ANCA levels should be examined in cases of aPAP complicated by pulmonary fibrosis.

It is generally thought that autoimmune diseases are induced by dysfunctional immunotolerance to self-antigens due to genetic as well as environmental factors [17]. Clinical evidence shows that the coexistence of autoimmune diseases within an individual (i.e., polyautoimmunity) is not uncommon, suggesting that these autoimmune diseases have a common genetic or environmental root [18]. It is also reported that aPAP sometimes accompanies other systemic or organ-specific autoimmune diseases. Seymour et al. reviewed 410 cases of PAP and reported seven cases (1.7%) with coexisting autoimmune disorders or positive autoimmune serology (rheumatoid arthritis, two cases; anti-smooth muscle antibody positive, two cases; multiple sclerosis, one case; IgA nephropathy, one case; and celiac disease, one case) [19]. Inoue et al. reported three cases among 212 patients with aPAP that were complicated by other autoimmune diseases including polymyalgia rheumatica, GPA, and autoimmune hemolytic anemia [2]. Whether the coexistence of aPAP and ANCA-associated systemic vasculitis in our case was anecdotal or due to shared underlying mechanisms remains to be elucidated. However, it is known that air pollution and/or infections sometimes overwhelm immunotolerance to MPO, even in healthy individuals [20]. Owing to their genetic tendency toward autoimmune disease and the altered immunologic milieu in their lungs, aPAP patients might be prone to developing an autoimmune reaction to MPO, triggered by an unknown environmental factor.

## Conclusion

Previous research has not succeeded in explaining why pulmonary fibrosis occurs in patients with aPAP, or how we should treat this complication, which is often associated with a poor prognosis. This is the first case report to suggest a pathogenetic relationship between ANCA-associated systemic vasculitis and aPAP-related pulmonary fibrosis. The link between ANCA-associated systemic vasculitis and aPAP-related pulmonary fibrosis must be explored with further research.

## Consent

Because the patient passed away due to the progression of respiratory failure, written informed consent was obtained from the patient’s wife for publication of this case report and any accompanying images. A copy is available for review by the Editor of this journal.

## Abbreviations

aPAP: Autoimmune pulmonary alveolar proteinosis
GM-CSF: Granulocyte-macrophage colony stimulating factor
HRCT: High-resolution computed tomography
BALF: Broncho-alveolar lavage fluid
MPO: Myeloperoxidase
ANCA: Anti-neutrophil cytoplasmic antibody
MPA: Microscopic polyangiitis
GPA: Granulomatosis with polyangiitis.
